# The Israeli eye care system through a public and global health lens

**DOI:** 10.1186/s13584-025-00731-2

**Published:** 2025-12-08

**Authors:** Mattan Arazi, Marcia Zondervan, Asaf Israeli, Hadash Maoz, Cova Bascaran Fanego

**Affiliations:** 1 Goldschleger Eye Institute, Sheba Medical Center, Tel Hashomer, Ramat Gan, Israel; 2 Faculty of Medicine, Tel Aviv University, Tel Aviv, Israel; 3 London School of Hygiene & Tropical Medicine, International Centre for Eye Health, Keppel Street, London, WC1E 7HT UK; 4 Vanderbilt Eye Institute, Vanderbilt University Medical Center, Nashville, Tennessee United States of America; 5 The Optometrists’ Council in Israel, Tel Aviv, Israel

**Keywords:** Israel, Eye health, Global eye health, Universal health coverage, Eye care

## Abstract

Global eye health is increasingly recognized as integral to achieving universal health coverage (UHC), with equity, access, and service integration serving as key indicators of system performance. Israel provides a distinctive case study, having established UHC through the 1994 National Health Insurance Law, which guarantees essential services to all residents. This review assesses Israel’s eye care system through a public and global health lens, applying the WHO health system building blocks to examine its organization, strengths, and persistent challenges. Israel benefits from strong integration of eye care within its universal framework, a high density of specialists, and wide availability of advanced services; yet inequities remain, particularly for lower-income and peripheral populations who face financial and geographic barriers to advanced treatment and consistent follow-up. Priority reforms include strengthening primary and community-based services, redistributing the workforce, and adopting nationally representative data and global indicators such as effective cataract surgical coverage (eCSC). Israel’s experience highlights both the potential and limits of universalism, offering lessons for countries seeking to integrate eye health into broader health planning as part of the global drive toward UHC.

**Supplementary Information:**

The online version contains supplementary material available at 10.1186/s13584-025-00731-2.

## Background

Eye health and vision care are fundamental components of public health. Globally, an estimated 2.2 billion people live with visual impairment, and at least 1 billion of these cases could have been prevented or treated [1]. Beyond its clinical impact, vision loss reduces quality of life, constrains workforce participation, limits educational attainment, and deepens poverty [2, 3]. Given its scale, socioeconomic consequences, and the availability of cost-effective interventions, eye health serves as a sensitive marker of health system performance and progress toward universal health coverage (UHC), the global movement to ensure access to essential services without financial hardship [3, 4].

Israel provides an instructive case study. Since the enactment of the National Health Insurance Law in 1994, all residents have been entitled to a comprehensive package of services under a universal framework [5, 6]. Eye care is an integral part of this framework, with key services included in the public health benefits package, offering a unique perspective for evaluating health system performance and equity across the country.

In this analytic review, we (i) summarize global developments in eye health, (ii) examine Israel’s eye care system using relevant elements of the World Health Organization (WHO) health system building blocks framework, and (iii) propose policy priorities to strengthen eye health within the country’s universal health coverage framework.

## Global developments in eye health

Globally, vision loss disproportionately impacts low- and middle-income countries (LMICs), which bear 90% of the burden [3]. Within countries, women, older adults, poorer households, and rural or minority populations remain at greater risk of preventable or treatable vision impairment [3, 7]. In high-income countries (HICs), chronic age-related conditions such as diabetic retinopathy, glaucoma, and age-related macular degeneration (ARMD) dominate. Low- and middle-income countries (LMICs) contend with a dual burden of untreated cataracts, uncorrected refractive error, and communicable eye diseases, driven by persistent limitations in health infrastructure [7–9]. The global economic impact is substantial, with vision impairment estimated to cost $411 billion annually [2].

Over the past two decades, international initiatives have shifted from vertical programs targeting specific eye diseases toward integrated, people-centered approaches aligned with health system strengthening. The WHO’s *World Report on Vision* (2019) and later World Health Assembly (WHA) resolutions emphasize the need to embed eye health within universal health coverage (UHC), with primary eye care, financial protection, and equity as central priorities [1, 10, 11]. Yet despite this momentum, major challenges persist. Eye health is still under-prioritized by many national policies, with limited integration of primary eye care and insufficient investment [12]. Moreover, population aging is driving an absolute increase in vision loss, with 1.8 billion people projected to be affected by 2050 [13].

These gaps highlight the importance of assessing national systems through a global health lens. Israel, with its model of UHC, provides a distinctive high-income case study for examining how international strategies and unresolved challenges manifest within a mature health system.

## Demographic overview

### Demographic trends and implications for eye health

Israel’s population is projected to reach 13 million by 2050, with life expectancy among the highest in the OECD [14]. The share of adults aged 65 and older is growing at twice the rate of the total population, and those aged 75 and above have expanded more than thirty-fold since the 1950 s [15]. This rapid aging is expected to substantially increase the prevalence of chronic eye disease, underscoring the need for robust health service planning in the eye health sector. Despite strong health outcomes overall, Israel’s health expenditure per capita and as a proportion of GDP remain below OECD averages [14, 16], which may reflect a relatively efficient system, though it also raises questions about the capacity to meet future needs.

### Prevalence and causes of vision impairment

National data on vision impairment remain limited. The National Registry for the Blind (NRB) provides annual incidence and etiology of blindness, with completeness supported by incentives such as rehabilitation benefits and financial allowances [17]. However, it captures only legal blindness and excludes milder impairment, restricting its use for comprehensive surveillance. Modelled global estimates suggest prevalence levels similar to other high-income countries, although Israel’s aging population may contribute to higher crude rates [18, 19]. Registry data indicate that age-related macular degeneration (ArMD), glaucoma, and inherited retinal disease are the leading causes of blindness, with diabetic retinopathy and cataract contributing smaller shares [17]. Incidence of major causes declined from 1999 to 2014 but has since plateaued, apart from diabetic retinopathy, which continues to decrease slowly. These patterns reflect the benefits of earlier system investments but also highlight the growing pressures of demographic aging. The absence of population-based surveys limits the accuracy of estimates and may hamper effective planning and monitoring.

### Projected cataract burden

Cataract, the leading global cause of blindness, is expected to rise sharply in Israel with demographic aging. Projections suggest that the number of people affected will nearly triple between 2010 and 2050, particularly among those aged 80 and older [20]. Detailed figures are provided in Supplementary Table 1. Meeting this demand will require sustained expansion of surgical capacity and forward-looking service provision planning.

### Childhood blindness and pediatric eye health

Registry data indicate that most childhood blindness in Israel is attributable to inherited or developmental conditions such as retinal dystrophies, optic nerve anomalies, and cerebral visual impairment, whereas preventable causes including retinopathy of prematurity, cataract, and glaucoma are relatively uncommon [21, 22]. These patterns likely reflect the strengths of Israel’s neonatal and pediatric care systems, where screening programs and integrated pediatric eye care have reduced avoidable blindness [22, 23]. Yet the rising burden of inherited dystrophies, partly associated with consanguinity in some populations, underscores the need for genetic counseling and targeted health education [21, 24]. Notably, the registry records higher certification rates among boys than girls, a disparity not observed in other high-income country registries [21]. These disparities may stem from unequal care-seeking, referral, or certification practices, potentially leaving girls with untreated impairment and excluding them from related benefits.

### Governance and healthcare financing

The Ministry of Health (MOH) sets national health policy and regulates service provision. Since the 1994 National Health Insurance Law, all residents must enroll in one of four non-profit health maintenance organizations (HMOs), which compete but operate under a unified framework. Each provides a publicly funded “health basket” of services, including medications, equipment, procedures, and technologies, updated annually by a joint MOH–Ministry of Finance committee with stakeholder input [6, 25].

### Health insurance coverage and out-of-pocket expenditures

While universal enrollment ensures broad access, most citizens purchase additional coverage. Over 80% hold supplementary HMO insurance and roughly half of the population also carry private policies [26, 27]. Out-of-pocket spending accounts for 21% of total health expenditure, higher than the regional average, and nearly 6% of households face catastrophic health costs [28, 29]. Much of this relates to gaps in coverage and limits of voluntary health insurance. Data specific to eye care are not reported, but the structure suggests that patients without supplementary or private insurance are more exposed to financial burden.

### Equity and disparities

Despite universal coverage, marked inequalities persist. Expanded insurance is concentrated among higher-income groups, with Jewish populations more than twice as likely as Arab populations to hold such coverage [26, 30]. These disparities leave residents of peripheral regions and low-income households at greater risk of unmet need. Migrant workers and asylum seekers, estimated at nearly 80,000 in 2017, remain largely outside the public system [36]. Such disparities are likely to translate into financial and geographic barriers to eye care, but robust data to substantiate this link are lacking.

### Eye care benefits and co-payments

Core ophthalmic services are included in the national health basket, though co-payments apply for consultations, surgeries, and some medicines. Cataract surgery is publicly funded with a standard monofocal intraocular lens. Toric lenses remain outside the basic basket. A 2016 reform briefly required patients choosing a toric lens to pay the full cost privately, but subsequent adjustments restored public coverage of the surgery while retaining a fixed patient contribution for the lens [31]. Second-line therapies for ARMD and diabetic retinopathy may also entail substantial costs without extended coverage [26]. Eyeglasses are generally excluded, with subsidies available primarily for children and, in some cases, through supplementary insurance. A summary of services covered under the health basket and HMO plans is provided in Supplemental Table 2.

### Provider payment systems and reforms

Eye health providers in public hospitals in Israel are typically salaried, with additional fee-for-service (FFS) payments available for work performed outside regular hours, such as after-hours surgeries. Ophthalmologists in community settings, particularly in primary care, may be paid through fee-for-service or capitation agreements, depending on the specific HMO they are contracted with. Since 2010, Israel has implemented reforms, including expanding the use of Procedure-Related Group (PRG) payments, a system similar to the Diagnosis-Related Group (DRG) payment structure, which categorizes patients based on the treatment provided rather than the diagnosis [32]. These reforms mirror wider European efforts to increase efficiency and transparency, promote equity in hospital financing, and reinforce regulatory oversight by the Ministry of Health [32–34]. However, the specific impact of these provider payment systems on ophthalmology service delivery has yet to be thoroughly examined.

### The role of the private sector

The private sector—including hospitals, clinics, insurers, and technology firms—contributes not only to service delivery but also influences the public system and shapes the broader eye care landscape in Israel. Neoliberal health policies have encouraged public–private partnerships and fostered a dynamic startup ecosystem, expanding service availability, easing pressure on the public system, and accelerating the development and commercialization of advanced technologies [35–37]. Market forces can create competition that incentivizes quality improvement, as patients and commissioning bodies weigh providers based on service performance [38]. Yet reliance on private financing also risks deepening inequities. Growth in private services has led to “cream-skimming” practices, prioritization of profitable procedures, and fragmentation of care, while straining public hospitals [26, 39]. For example, private hospitals performing strabismus surgery were more likely to treat younger and less complex patients, likely to minimize complications and maximize revenue [40].

## Eye care workforce

The number of board-certified ophthalmologists in Israel has grown by about 1.3% annually over the past 15 years, reaching 90.6 per million people in 2021. This density is higher than in the United States and United Kingdom and comparable to France and Germany, while remaining below several European peers [41–44]. Despite this overall growth, subspecialist distribution remains uneven, with shortages in peripheral regions, particularly in retina care [26]. Gender parity has nearly been achieved among residents by 2015, although women remain underrepresented among senior specialists. The number of Arab ophthalmologists increased by 288% between 2006 and 2021, yet their overall share of the profession remains below their proportion of the population [45]. Alongside ophthalmologists, Israel has an estimated 2,500–2,880 licensed optometrists (263–294 per million) and about 120 certified orthoptists. Most work in private practice or retail settings, limiting their integration into the public health system [46, 47].

Cataract surgery, the most common ophthalmic procedure, provides a critical benchmark for assessing how workforce capacity translates into service access. In Israel, the procedure is broadly available in public hospitals, reflecting sustained investment in ophthalmic infrastructure. The Israeli Ophthalmological Society (IOS) reports a cataract surgical rate (CSR) of roughly 7,200 procedures per million annually, consistent with high-income country benchmarks [48, 49]. A global survey estimated that just over one third of Israeli ophthalmologists perform cataract surgery, though resident participation was not specified [44]. While these figures highlight the system’s ability to deliver high-volume surgery, CSR reflects volume alone and does not capture quality, outcomes, or unmet need. The absence of internationally endorsed indicators such as effective cataract surgical coverage (eCSC) and effective refractive error coverage (eREC) therefore represents a priority for strengthening monitoring and ensuring equity in access [49].

## Service provision

### Organization and accessibility

Primary eye care in Israel is provided through a combination of general practitioners, community ophthalmologists, and optometrists. Optometrists deliver refraction and low-vision services, but their limited integration into the public system reduces availability for patients dependent on publicly funded care. Community ophthalmologists act as both entry-point providers and specialists, with nationwide electronic medical records supporting continuity across clinics and hospitals [50]. Most hospitals operate ophthalmic emergency departments, ensuring rapid access to urgent care, though this model also risks overuse for non-urgent conditions. Waiting times vary by geography; for example, major centers offer specialist consultations within about two weeks, while smaller hospitals may face delays of six weeks or more [26, 51].

### Screening and preventive eye care

Israel has established screening programs across the life course. Neonatal red-reflex testing and systematic screening for retinopathy of prematurity support early detection of severe conditions [52, 53]. Preschool vision screening is nationally mandated, though compliance varies and only about half of children referred after preschool screening attend follow-up visits, largely due to parental and system barriers [21, 53]. Diabetic retinopathy screening is incorporated into the Quality Indicators in Community Healthcare program, with coverage improving modestly from 57% in 2002 to 63% in 2010, although more recent data are limited [54]. Mobile retinal imaging units have expanded access in peripheral regions. Screening for glaucoma and ARMD is recommended for high-risk populations, although adherence data are scarce [33]. Mandatory military service provides an additional layer of vision assessment at entry [55].

### Broader stakeholders

Beyond government and insurers, other stakeholders also contribute to Israel’s eye health system. Professional associations establish standards and support workforce training. Non-governmental organizations (NGOs) extend services to underserved groups. Israel’s research and innovation ecosystem, particularly in ophthalmic artificial intelligence and medical devices, has international reach. A fuller overview of these stakeholders is provided in the Supplementary Appendix. These contributions highlight the importance of broad engagement across sectors and set the stage for the policy recommendations that follow.

### Limitations

This review offers a broad overview of Israel’s eye care sector rather than an exhaustive critique of the wider health system. The scarcity of public health literature specific to eye care in Israel limited the depth of analysis and necessitated reliance on select reports, which may introduce bias. The focus on public hospitals excluded perspectives from private providers. Most importantly, there are no nationally representative data on the prevalence, incidence, risk factors, or outcomes of vision impairment. Evidence on service effectiveness, quality, and patient experience is also limited, constraining the ability to monitor trends or compare across subpopulations. These limitations constrain the ability to fully benchmark Israel against international indicators and highlight the urgent need for systematic, population-based data.

### Concluding remarks

Eye health must be recognized as a core component of population health [56]. Israel illustrates the benefits of embedding eye care within a universal framework: entitlements enshrined in the National Health Insurance Law, inclusion of major blinding conditions in the national health basket, and broad access to trained specialists and advanced services. Moreover, consistently high cataract surgical output demonstrates the system’s capacity to deliver essential procedures at scale. These achievements reflect a longstanding commitment to equity and have positioned the country close to global aspirations for universal eye health.

Yet persistent inequities highlight that universal coverage alone is not sufficient. Dependence on supplementary insurance, geographic maldistribution of subspecialists, and weak integration of optometry and community level services continue to shape access. Rapid population growth and demographic aging will intensify demand, underscoring the need to expand service capacity and improve workforce distribution. The absence of population-based surveys and internationally endorsed indicators such as eCSC and eREC further limits accountability and hampers benchmarking against global standards [49]. A fuller appraisal of strengths and challenges across the three dimensions of universal health coverage (population, service, and financial coverage) is presented in Supplemental Table 3. This framework highlights strong institutional foundations alongside gaps in equitable access, service continuity, and financial protection.

Israel’s experience offers lessons of wider relevance. Strong institutions and universal coverage can drive progress, but lasting gains will depend on sustained investment in equity, systematic monitoring, and community-based care to meet demographic pressures and ensure benefits are shared across all groups.

### Policy recommendations

A focused set of reforms can consolidate Israel’s achievements and close remaining gaps (Table 1). Priorities include establishing a national strategy with transparent reporting, adopting internationally comparable indicators, expanding surgical and community capacity, strengthening referral pathways, and ensuring more equitable workforce distribution and financing. Collectively, these measures would enable Israel to move from a system oriented toward advanced disease treatment to one that actively promotes eye health across the life course. With such reforms, Israel could not only secure equity and sustainability domestically but also serve as a model for integrating eye health into national health systems globally.

**Table 1:** Policy recommendations for strengthening eye care in Israel

**Priority area**	**Recommendation**
**National strategy and accountability**	Establish a national eye health strategy with transparent reporting to unify existing initiatives, align public and private actors, and strengthen accountability. Regular publication of service use and outcomes, disaggregated by region and population group, would enable equity-oriented planning and oversight
**Data and indicators**	Implement internationally endorsed metrics such as effective cataract surgical coverage (eCSC) and effective refractive error coverage (eREC). Supplement registry data with periodic population-based surveys to generate robust evidence, support international comparability, and guide targeted investment
**Demographic ageing and chronic eye disease**	Expand surgical and treatment capacity to address the rising burden of age-related eye conditions. Monitoring outcomes with eCSC and related measures will ensure that expanded services deliver high-quality, equitable care
**Screening and continuity of care**	Link screening programs more effectively to treatment. National referral-completion targets, supported by coordination between primary care, optometry, and tertiary services, would close persistent gaps in follow-up and reduce avoidable vision loss
**Primary care integration**	Expand the role of primary and community providers in managing routine eye conditions at the first point of contact. Embedding eye care within primary care pathways would reduce unnecessary referrals, enhance local capacity, and create a more balanced system less dependent on hospital-based services
**Workforce planning and distribution**	Address geographic maldistribution of subspecialists and strengthen the integration of optometry within the public sector. Strategic deployment and multidisciplinary collaboration would ensure that both specialist and primary eye care are equitably available nationwide
**Financing and equity**	Ensure predictable coverage for advanced therapies and refractive correction, while reducing reliance on supplementary and private insurance to protect households from financial strain. Strengthening provider payment models to reward quality and efficiency would further support accountability and timely access

## Supplementary Information

Additional file 1.

## Data Availability

No datasets were generated or analysed during the current study.
