# Supplementary material for: The Israeli eye care system through a public and global health lens
Source: Isr J Health Policy Res. 2025 Dec 8;14:74. doi: 10.1186/s13584-025-00731-2 (PMC12683923; doi:10.1186/s13584-025-00731-2)
Supplement: Supplementary file 1 — Additional file 1. [file 13584_2025_731_MOESM1_ESM.docx]

**Supplementary Table 1. Projected Growth in Cataract Prevalence by Age Group: Trends in Israel from 2010 to 2050**

To approximate the project burden of cataract in 2050, we used data from the National Eye Institute (NEI)^1^ as a proxy, illustrating the projected increase in cataract prevalence across age groups (Supplementary Table 1). While NEI estimates are based on the U.S. population and may not fully reflect Israel’s demographic and healthcare characteristics, they provide a useful reference in the absence of national cataract prevalence data. We acknowledge that variations in risk factors such as diabetes, myopia, and smoking between populations may impact cataract prevalence, making direct comparisons imperfect. However, given the lack of more regionally relevant data, these estimates serve as a reasonable basis for assessing proportional trends in cataract burden and service provision planning.

Our projections indicate that the number of individuals with cataracts in Israel will increase by approximately 195% between 2010 and 2050 and by 73% between 2030 and 2050. The most significant proportional rise will be among individuals aged 80 and older, reflecting broader global patterns of aging and disease burden. These projections underscore the growing need for expanded cataract surgery capacity and scalable service provision in Israel.

| **Age Group** | **Age-Specific Prevalence of Cataract (%)** | **Population in 2010** | **Individuals with Cataract in 2010** | **Population in 2030** | **Individuals with Cataract in 2030** | **Population in 2050** | **Individuals with Cataract in 2050** | **Proportionate Increase (2010-2050) (%)** | **Proportionate Increase (2030-2050) (%)** |
| --- | --- | --- | --- | --- | --- | --- | --- | --- | --- |
| 50–54 | 5.2 | 358,237 | 18,700 | 540,182 | 28,198 | 678,708 | 35,429 | 89.5 | 25.6 |
| 55–59 | 9.1 | 344,343 | 31,473 | 499,079 | 45,616 | 624,099 | 57,043 | 81.2 | 25.1 |
| 60–64 | 15.5 | 295,512 | 45,657 | 429,660 | 66,382 | 587,555 | 90,777 | 98.8 | 36.8 |
| 65–69 | 24.7 | 208,161 | 51,478 | 368,045 | 91,018 | 549,446 | 135,878 | 164.0 | 49.3 |
| 70–74 | 36.5 | 172,543 | 62,961 | 324,756 | 118,503 | 509,296 | 185,842 | 195.2 | 56.8 |
| 75–79 | 49.5 | 150,527 | 74,496 | 285,221 | 141,156 | 442,162 | 218,826 | 193.7 | 55.0 |
| 80+ | 68.3 | 201,673 | 137,743 | 335,107 | 228,878 | 763,011 | 521,137 | 278.3 | 127.7 |
| **Total** |  | 1,730,996 | 422,507 | 2,782,050 | 719,751 | 4,154,277 | 1,244,931 | **194.7** | **73.0** |

Note: Cataract prevalence was extracted from data provided by the National Eye Institute (NEI) as a proxy.^1^ Population data for Israel was obtained from the International Database of the U.S. Census Bureau.^2^

**Supplementary Table 2.** Summary of Eye Care Services and Coverage Under Israel’s Health Basket and HMOs

| **Category** | **Service** | **Covered by Health Basket** | **HMO Coverage*** | **Key Notes** |
| --- | --- | --- | --- | --- |
| **Primary Eye Care** | **Optometry Services** | No | Partial | Increased coverage with extended insurance |
|  | **General Practitioner** | Yes | Full | Basic eye care (conjunctivitis, simple referrals). |
| **Secondary/Tertiary Care** | **Comprehensive Eye Exams & Screening** | Yes | Full | Annual check-ups free; extra exams may incur cost. Mostly in community clinics. |
|  | **Pediatric Eye Clinic Visits** | Yes | Full for children (<18) | Extended coverage for developmental conditions. |
|  | **Ophthalmology Subspecialties** | Yes | Full | Varies by subspecialty (retina, glaucoma, cornea, etc.). |
|  | **Ophthalmic ER/ED Visits** | Yes | Full | Non-emergency visits can mean out-of-pocket costs. Certain urgent procedures and referrals exempt. |
|  | **Hospital Admissions** | Yes | Yes |  |
| **Surgeries** | **Standard Cataract Surgery** | Yes | Yes | Includes preliminary tests. Waiting periods may apply. |
|  | **Premium Intraocular Lens** | No | Surgery covered, lens not | Premium/multifocal lens cost often out-of-pocket/extended insurance plans. |
|  | **Refractive Surgery (e.g., LASIK)** | No | Partial (specific criteria) | Covered if medically necessary (severe anisometropia). |
|  | **Vitrectomy** | Yes | Full | Includes pre- and post-op care. |
|  | **Glaucoma Surgery** | Yes | Full | Advanced techniques may require extra payment. |
| **Vision Aids** | **Contact Lens Fitting** | Yes (medically indicated) | Up to age 18 (specific needs) | Fully covered for keratoconus or similar. |
|  | **Eyeglasses** | Yes | Up to 18 | Children’s glasses often fully covered once a year. |
| **Low Vision** | **Refractive testing, optical and non-optical assistive devices, rehabilitation** | Partial | Partial: vision exams and rehabilitation | Optical (magnifiers, telescopic glasses) and electronic aids (screen readers, Braille devices) not in the health basket but may be partially subsidized through rehabilitation programs and extended insurance. |
| **Diagnostic Exams** | **Orthoptics** | Yes | Full | Follow-up covered, especially for children with strabismus. |
|  | **Perimetry** | Yes | Full | Used for glaucoma & neurological assessments. |
|  | **Optical Coherence Tomography (OCT)** | Yes | Full | Common for retinal checks; may have partial limits in practice. |
| **Preventive** | **Preliminary Tests for Surgery (Imaging, etc.)** | Yes | Full | Ultrasound, anterior segment imaging, etc. |
|  | **Diabetic Retinopathy Screening** | Yes | Full | . |
| **Additional Services** | **Genetic Testing** | Yes | Full | Only certain hereditary conditions may get coverage. |
| **Special Circumstances** | **Coverage for Soldiers** | Covered by Ministry of Defense | Full for authorized services |  |
| **Medications** | **Specific Eye Medications** | Yes | Typically, Full | High-cost meds (e.g., specific anti-VEGF) have coverage caps. |

***Policy Dependent:** In Israel, the coverage of medical services is determined by the National Health Basket and individual health fund policies and may be case-dependent. Treatments included in the Health Basket and provided within public hospitals or approved facilities are typically fully covered. Services through contracted hospitals or specialists are generally covered, though co-payments may apply depending on clinical criteria and institutional agreements. Partial coverage or out-of-pocket expenses may be required for elective procedures, such as premium intraocular lenses or refractive surgery, and for treatments sought outside the health fund’s approved network. Certain high-cost medications, including specific anti-VEGF therapies, may necessitate supplemental insurance or direct patient payment, with coverage contingent on medical necessity and health fund policies.

**Supplemental Table 3:** Strengths and Challenges of Israel’s Eye Health System in Achieving Universal Health Coverage

| Dimension | Strengths | Challenges |
| --- | --- | --- |
| Population Coverage | -Strong integration of eye care within Israel’s broader UHC framework ensures coverage for major blinding conditions across the life course.  - Universal mandatory enrollment in one of four non-profit HMOs ensures that all legal residents have access to a broad range of essential eye care services.  - High density of ophthalmologists (103–124 per million people) ensuring broad availability of specialized care.  -Comprehensive pediatric screening programs (e.g., neonatal red reflex screening), mandatory military medical screening for young adults, and diabetic retinopathy screening for at-risk patients  -The National Blind Registry (NRB) likely captures the majority of individuals with blindness in Israel, providing accurate estimates and comprehensive data on the blindness burden.  -Introduction of reforms to improve access to specialized treatments, such as Toric IOLs, enhances equity in eye care for broader population groups  -Designated “Health Days” run by the HMO’s and mobile eye clinics in peripheral parts of the country increase access to screening and treatment | - Rapidly aging population expected to increase the burden of age-related eye diseases (e.g., glaucoma, ARMD, cataracts), requiring enhanced service capacity and long-term planning  - Patient adherence to screening for specific eye conditions (diabetic retinopathy, preschool vision screening) is not optimal, potentially resulting in the missed detection of sight-threatening but preventable conditions.  -Inconsistent implementation of national guidelines for routine glaucoma and ARMD screening in at-risk adults may contribute to potential under-detection of these conditions.  - Non-citizen populations face barriers in accessing public eye care services.  - Limited data on the geographic distribution of eye care personnel makes it more difficult to identify and address local disparities in access.  - Lack of large-scale, population-based surveys to assess the true national prevalence and causes of vision impairment  - While eye care is well integrated into broader health policies, the absence of a dedicated public eye health strategy presents an opportunity to enhance coordination, outcome tracking, and alignment with global indicators. |
| Service Coverage | - Israel’s national health basket includes essential eye care services, such as cataract surgery, diabetic retinopathy treatment, and glaucoma care  - Cataract surgery and retina services (intra-vitreal injections, laser treatment) provided by most hospitals in the country  - Advanced ophthalmic services, including ocular oncology, complex vitreoretinal surgery, and diagnostic imaging, are available at tertiary referral centers.  - Integration of primary eye care into general healthcare through GP referrals and optometrists, enabling early detection and streamlined referrals.  - Strong CSR of approximately 7,200 surgeries per million annually, comparable to other high-income countries.  -National diabetic retinopathy and child vision screening programs support early detection and have strengthened preventive care.  - A nationally integrated electronic medical records system connects hospitals and outpatient clinics, enhancing continuity of care and referral efficiency.  - Active engagement of stakeholders, including HMOs, private providers, and NGOs, supporting service delivery and innovation. | -Limited-service coverage for non-referred vision correction (e.g., prescription glasses), cosmetic procedures (e.g., oculoplastics), and refractive surgery  - Wait times may be longer in smaller, peripheral hospitals, pointing to opportunities to improve geographic equity in service delivery.  - Limited availability of national data on Cataract Surgical Coverage (CSC) and effective Cataract Surgical Coverage (eCSC), making it difficult to assess service quality and impact and unmet needs.  - Easy referral to ophthalmic emergency departments may overstrain health system with non-urgent cases, as well as encourage consumer moral hazard.  - “Cream skimming” practices in private services over refer complicated cases to public hospitals |
| Financial Coverage | - Most essential eye care services are fully or partially covered by the NHIL, reducing financial barriers for residents with public or supplementary insurance.  - Supplementary insurance plans offered by HMOs provide partial coverage for specialized services, reducing costs for those who can afford additional coverage.  - Reforms in financing models, such as the Procedure-Related Group (PRG) payment system, improve transparency and efficiency in hospital-based care.  - Access to cost-effective generic medications, including ophthalmic drugs, through local pharmaceutical production (e.g., Teva Pharmaceuticals) helps reduce treatment costs for common conditions. | - High co-payment costs associated with advanced treatments (e.g., anti-VEGF therapy for ARMD, second-line diabetic retinopathy treatments) may restrict access for underinsured patients or those who are not covered by HMO policies.  - Socioeconomic disparities in supplementary insurance coverage: higher-income and the Jewish population are more likely to have expanded coverage compared to lower-income and the Arab population.  - Approximately 20% of total health expenditures are out-of-pocket, higher than some other HICs with UHC (e.g., the United Kingdom and Germany), posing financial strain on low-income households.  - Private sector dominance in high-cost services (e.g., cosmetic surgery, advanced refractive procedures) contributes to inequities, with lower-income populations relying solely on public services  -Prescription glasses are taxed as a commodity and typically not covered under standard health plans, which may pose affordability challenges for some. |

**Note:** ARMD = age-related macular degeneration; HMO = Health Maintenance Organization; GP = General Practitioner; CSR = Cataract Surgical Rate; NHIL = National Health Insurance Law; ARMD = Age-Related Macular Degeneration; VEGF = Vascular Endothelial Growth Factor; HIC = High-Income Country; UHC = Universal Health Coverage

**Stakeholders in the Israeli Eye Care Sector**

The Israeli eye care sector involves a wide constellation of actors beyond governmental bodies and health facilities. Professional associations, nongovernmental organizations (NGOs), academic institutions, and private industry each contribute to shaping services, research, advocacy, and innovation. Together they complement the universal system by addressing unmet needs, advancing research, promoting public awareness, and expanding access to emerging technologies.

**Professional organizations** such as the Israeli Ophthalmological Society (IOS) and the Optometrists’ Council in Israel (MAI) set professional standards, represent providers in policy forums, and promote education and workforce development. The Israeli Society for Vision and Eye Research (ISVER), affiliated with the Association for Research in Vision and Ophthalmology (ARVO), fosters collaboration and supports high-quality research.

**Nongovernmental organizations (**NGOs) provide services to underserved populations and advocate for blindness prevention. Examples include the Center for the Blind in Israel, which coordinates low-vision services; St. John’s Eye Hospital, which delivers essential care to Palestinians in the West Bank and Gaza;^3^ Sheba Global Ophthalmology, which leads international capacity-building initiatives;^4^ and the School of Optometry and Vision Science at Bar-Ilan University, which runs mobile outreach clinics across rural communities and care facilities. ^5^

**Research, innovation, and industry.** Israel has a vibrant research culture, with strong global output in ophthalmology. ^6^ It is also recognized for advances in artificial intelligence and medical technology, such as BELKIN Vision’s automated laser for glaucoma and AEYE Health’s FDA-approved AI screening for diabetic retinopathy. ^7,8^ The pharmaceutical sector, led by Teva, contributes by expanding access to affordable generics, including ophthalmic drugs. ^9^

**References**

1. Cataract tables. National Institutes of Health. National Eye Institute. February 7, 2020. Accessed February 18, 2025. https://www.nei.nih.gov/learn-about-eye-health/eye-health-data-and-statistics/cataract-data-and-statistics/cataract-tables

2. International Data Base (IDB). U.S. Census Bureau. 2010. Accessed February 18, 2025. https://www.census.gov/data-tools/demo/idb/#/dashboard

3. St. John of Jerusalem Eye Hospital Group . Accessed October 19, 2024. https://www.stjohneyehospital.org/donate-now/emergency-appeal/

4. Sheba Global Ophthalmology. . Accessed October 19, 2024. https://sheba-global.com/ophthalmology/

5. *Bar-Ilan Report: 2023 Final Report. European Council of Optometry and Optics (ECOO)*.; 2023. Accessed February 6, 2025. https://ecoo.info/wp-content/uploads/2023/11/Bar-Ilan-Report-Final-July-2023.pdf

6. Assayag E, Mimouni M, Bettach E, Zadok D, Abulafia A, Weill Y. [PUBLICATION TRENDS AND IMPACT OF OPHTHALMOLOGY DEPARTMENTS IN ISRAEL: SUMMARIZING OVER A DECADE OF RESEARCH WITH AN INTERNATIONAL PERSPECTIVE (2011-2021)]. *Harefuah*. 2024;163(3):145-150.

7. Joy Jordana. AEYE Health receives FDA clearance for first ever fully autonomous AI for portable DR screening. . Optometry Times. . May 2, 2024. Accessed January 4, 2025. https://www.optometrytimes.com/view/aeye-health-receives-fda-clearance-for-first-ever-fully-autonomous-ai-for-portable-dr-screening

8. Takusagawa HL, Hoguet A, Sit AJ, et al. Selective Laser Trabeculoplasty for the Treatment of Glaucoma: A Report by the American Academy of Ophthalmology. *Ophthalmology*. 2024;131(1):37-47. doi:10.1016/j.ophtha.2023.07.029

9. Teva Announces Launch of First Generic Version of AZOPT® (brinzolamide ophthalmic suspension) 1%, used to treat high pressure inside the eye, in the United States. Teva. . March 8, 2021. Accessed January 4, 2025. https://www.tevapharm.com/news-and-media/latest-news/teva-announces-launch-of-first-generic-version-of-azopt-brinzolamide-ophthalmic-suspension-1-used-to/
